# Walking with Music Is a Safe and Viable Tool for Gait Training in Parkinson's Disease: The Effect of a 13-Week Feasibility Study on Single and Dual Task Walking

**DOI:** 10.4061/2010/483530

**Published:** 2010-07-13

**Authors:** Natalie de Bruin, Jon B. Doan, George Turnbull, Oksana Suchowersky, Stephan Bonfield, Bin Hu, Lesley A. Brown

**Affiliations:** ^1^Department of Kinesiology, University of Lethbridge, 4401 University Drive, Lethbridge, AB, Canada T1K 3M4; ^2^Faculty of Health Professions, Dalhousie University, Halifax, NS, Canada B3H 1R2; ^3^Department of Clinical Neurosciences, University of Calgary, 3330 Hospital Drive NW, Calgary, AB, Canada T2N 4N1; ^4^Department of Medical Genetics, University of Calgary, Calgary, AB, Canada T2N 4N1

## Abstract

This study explored the viability and efficacy of integrating cadence-matched, salient music into a walking intervention for patients with Parkinson's disease (PD). Twenty-two people with PD were randomised to a control (CTRL, *n* = 11) or experimental (MUSIC, *n* = 11) group. MUSIC subjects walked with an individualised music playlist three times a week for the intervention period. Playlists were designed to meet subject's musical preferences. In addition, the tempo of the music closely matched (±10–15 bpm) the subject's preferred cadence. CTRL subjects continued with their regular activities during the intervention. The effects of training accompanied by “walking songs” were evaluated using objective measures of gait score. The MUSIC group improved gait velocity, stride time, cadence, and motor symptom severity following the intervention. This is the first study to demonstrate that music listening can be safely implemented amongst PD patients during home exercise.

## 1. Introduction

The gait disturbances that characterise Parkinson's disease (PD) have been associated with increased fall risk, diminished mobility [1, 2], loss of independence [1, 3], and reduced quality of life [4, 5]. Mobility impairments and fall risk amongst PD patients are further exacerbated when patients are engaged in a secondary task [6–11], such as talking whilst walking. This phenomenon, known as dual task interference, is considered a common contributing factor to falls in the elderly, especially those with movement disorders and/or dementia [12]. The inability to consistently manage gait deficits with pharmacological treatments has led to the development of rehabilitation strategies intended towards relieving gait impairments. One rehabilitation strategy that has frequently been reported as effectual for improving gait performance in PD is the use of rhythmic auditory cues [13–17]. Single session studies have established the effectiveness of auditory cueing in temporarily improving gait velocity, amplitude, frequency, and variability across single [13, 15] and dual task [16, 17] contexts. Furthermore, a number of studies have demonstrated the feasibility of incorporating auditory cues into rehabilitation strategies to significantly improve gait performance and decrease motor symptom severity [14, 18–23]. Although the efficacy of training with a simple repetitive tone [14, 18–20, 22] or rhythmically accentuated music [21, 23] to facilitate parkinsonian gait has been widely documented, practical applications and the benefits of these rehabilitation strategies are constrained by several factors. First, there has been a lack of studies investigating how a musical piece or “walking song” should be constructed for individual patients thereby optimising the congruence of the music with walking and exercise and maximizing the positive cueing effect. Second, even though the use of music at home during exercise may be considered beneficial and highly desirable, safety remains a major concern for patients. Previously, we reported that listening to music may have a distractive effect when initially combined with walking, possibly creating a dual task condition [24]. It is possible, however, that this is a short-term phenomenon and that over time patients may become accustomed to the task. 

To address these issues, in this study we implemented a 13-week home-based music and walking program. We selected commercially available music that was unaltered and familiar and enjoyable to individual patients. The tempo of each musical piece was carefully evaluated to ensure that it closely matched the preferred walking cadence of the respective patient. The temporal matching contrasts the aforementioned study, in which the intrinsic properties of the music were not controlled, potentially contributing to the gait deficits demonstrated by PD patients whilst walking with music [24]. In the current study spatiotemporal parameters of gait and symptom severity were assessed pre- and post-intervention. Based on the substantiated effectiveness of rhythmic auditory cues in producing immediate and short-term improvements in parameters of gait across a variety of functional gait activities [13–23] we hypothesized that a walking intervention that incorporated a music cueing program could be safely implemented and would result in improved gait performance across single and dual task test conditions post-intervention.

## 2. Methods

### 2.1. Subjects

Thirty-three patients with mild to moderate PD were enrolled from two research centres: University of Lethbridge, Lethbridge, Canada (*n* = 12) and Dalhousie University, Halifax, Canada (*n* = 21). Following enrolment, patients were randomly allocated to a control (CTRL) group (*n* = 17) or an experimental (MUSIC) group (*n* = 16). The Lethbridge subjects provided data (Unified Parkinson's Disease Rating Scale (UPDRS [25]), Gait and Balance Scale (GABS [26]), Activities-specific Balance Confidence scale (ABC [27]), and Parkinson's Disease Questionnaire-39 (PDQ-39 [28])) for a companion study to be reported subsequently. The GABS, ABC, and PDQ-39 data are not included in this study. 

Eligibility criteria were diagnosis of PD (United Kingdom Brain Bank Criteria [29]), stage II-III on the Hoehn and Yahr scale [30], stable medication regimen, independently mobile without the use of a walking aid, and intact hearing. Patients were excluded from the study if diagnosis was less than one year, if they had undergone deep brain stimulation surgery, if they experienced regular freezing episodes (self-report), or if they were unable to ambulate independently in the community. Exclusion criteria also included the presence of neurological disorders or comorbidities likely to affect gait, scoring 24 or less on the Mini-Mental Status Examination (MMSE [31]) and/or already walking with music.

### 2.2. Ethics Statement

The study was performed with approval by the University of Lethbridge Human Subject Research Committee and The Dalhousie University Health Sciences Research Ethics Board in accordance with the Declaration of Helsinki. All subjects were informed of the nature of the study and provided informed written consent prior to the start of the study.

### 2.3. Intervention

The CTRL group continued with any regular activities for the 13-week intervention period. The MUSIC group walked at least 30 minutes, three times a week at a comfortable pace whilst listening to an individualised music playlist through head/ear-phones on an iPod (Apple Inc., Cupertino, CA) in addition to maintaining regular activities. Subjects walked on their own in the community and were asked to refrain from dual tasking (i.e., conversing with companions or walking with pets) whilst participating in the music accompanied walks. Each of the subjects in the CTRL and MUSIC group maintained an “Activities and Falls” diary in which physical activities, activity duration, and any falls experienced were documented each day. All subjects were contacted biweekly to monitor and ensure compliance.

### 2.4. Pre- and Post-intervention Assessment

Outcome measures were assessed immediately prior to randomisation (pre-intervention) and after 13 weeks (post-intervention). Subjects were tested on medications, at the same time of day for pre- and post-intervention assessments. Subjects walked the length of a 10-metre walkway at a self-selected pace in six different test conditions. Test conditions were differentiated by the presence of music accompaniment (no music/music) and the requirement to perform a simultaneous cognitive task (single task/dual task) or negotiate a three-dimensional foam block obstruction (no obstacle/obstacle). This paper explores the training effects of a 13-week music accompanied walking program on single and dual task walking; therefore the results reported are for single and dual task walking trials without music. In addition, due to the differences in motor patterning between unobstructed and obstructed walking, the effects of the walking intervention on obstacle negotiation will be addressed in a separate paper. 

**Figure 1:**
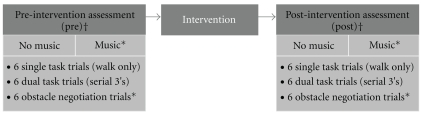
Experimental design. † Trials randomised. *Not included in current analysis.

The cognitive task consisted of serial 3 subtractions from a random 3 digit number. A new starting number was provided for each dual task trial immediately prior to trial commencement. Subjects were instructed to prioritise walking and the cognitive task equally. Subjects completed 6 trials in each condition (*N* = 36 trials; Figure 1). Task presentation was randomised to control for order and practice effects. Music conditions were counterbalanced between subjects. One practice trial was performed for each task prior to the start of the testing session. A trained researcher supervised all subjects during testing to ensure subject safety. Frequent rests were provided to avoid fatigue.

### 2.5. Music

The cadence of each subjects' preferred walking speed was determined during the screening visit. The length of the walkway used to determine preferred cadence was 10 metres. Following randomisation, MUSIC subjects participated in a telephone interview with a music specialist (S.B.), detailing their music listening habits and preferences. An individualised music playlist was created for each subject with a specific arrangement of tempo-to-cadence-matched songs that were identified to be salient to the patient. The music specialist defined music salience based on genre, artist, and song preferences. The range of tempos for each playlist closely matched (±10–15 beats per minute) the preferred walking cadence of each respective patient. The tempo of each piece was determined independently by two raters using a metronome; agreement between raters was absolute. Each playlist was loaded to a personal music player (iPod Nano or Shuffle based on personal preference) and subjects were offered a choice of earbuds or headphones to maximize comfort. Playlists were approximately one hour in duration, and subjects were asked to play through the playlist in the sequence provided rather than setting the music player in “shuffle” mode. Subjects were asked to refrain from listening to the music outside of the walking program. Prior to the commencement of the intervention subjects were provided with a familiarisation period on the iPod. The familiarisation period was concluded when the subject indicated confidence in operating the personal music player independently. Subjects were also informed that they could request changes to their playlist at any stage of the intervention.

### 2.6. Apparatus

Kinematic data were collected using the technology available at each research centre. Gait parameters were assessed using a 6.5-metre instrumented GAITRite mat (100 Hz; Dalhousie University; CIR Systems Inc, Havertown, PA) that was placed at the centre of a 10 m walkway or alternatively using a six camera motion analysis system (120 Hz; University of Lethbridge; Vicon-Peak, Peak Performances Technologies, Englewood, CO). Seventeen passive markers were placed on the subjects for use with the camera motion analysis system as previously described [24]. The validity and reliability of the GAITRite and Vicon systems for measuring spatiotemporal parameters of gait have previously been established [32]. In addition, the intersystem reliability has been determined, with intraclass correlation coefficients of between 0.92 and 0.99 indicating good agreement between the GaitRite and Vicon systems for averaged spatial and temporal gait data. Absolute differences between the systems were reported as 0.02 m/s, 2.03 steps/min, and 0.02 m for gait velocity, cadence, and stride length, respectively [33]. An iPod Nano with microphone, attached to the subjects' shirt, was used to capture verbalizations during the dual task trials.

### 2.7. Outcome Measures

Aligned with the main goal of many gait training interventions, the primary outcome measure for this study was gait velocity (m/s) with secondary outcome measures consisting of stride time (s), stride length (m), and cadence (steps per min). Error rates (subtraction errors: number of subtractions; %) on the dual task were also evaluated. Additionally, motor symptom severity was assessed using the UPDRS (III) motor section. All UPDRS (III) motor assessments were performed by a trained evaluator. The evaluator was blinded to subject group assignment; the order of assessment among groups was randomised within and between days. 

Descriptive measures obtained pre-intervention included the Modified Baecke Questionnaire for Older Adults [34] and MMSE [31]. An eight-item questionnaire was administered to the MUSIC group post-intervention to determine music and intervention tolerance and adherence. The “Activities and Falls” diary was used to determine compliance to the intervention, walk duration, activity level, and number and causes of falls during the intervention period.

### 2.8. Data Processing

Raw marker data collected using the camera motion analysis system were filtered at 10 Hz using a lowpass fourth-order Butterworth filter and then processed using custom written algorithms (MATLAB, Version R2007a; The Mathworks Inc, Redmond, WA). A seven-segment model was used to calculate location of whole body centre of mass (COM) in the anterior-posterior (AP) direction. The finite differences method was then used to determine AP COM velocity. Kinematic data were cropped into gait cycles using the event of right heel contact. Mean values across gait cycles were calculated for each trial and trials were averaged across each test condition for each subject. 

Verbal data collected during the dual task trials were scored manually to determine relative error rates, based on a ratio of the number of subtraction errors to the number of subtractions during the trial. Delayed responses were considered correct or incorrect as appropriate. Mean values were calculated pre- and post-intervention.

### 2.9. Statistical Analysis

Data were analysed using SPSS Statistics 17.0 for Windows (SPSS Inc., Chicago, IL). Subject characteristics, baseline measures, falls, and walk durations were summarised descriptively and compared between groups using independent *t*-tests or chi-square tests. Responses to the eight-item Post-intervention Questionnaire were summarized descriptively for the MUSIC group.

Disease severity could not be considered homogeneous between groups pre-intervention. Accordingly, separate 2-factor [Time (PRE/POST) × Task (SINGLE/DUAL)]. Repeated-Measures Analyses of Variance (RM-ANOVA) were used to establish the effect of time and task on primary outcome measures within each group. Significant interactions were followed up with paired *t*-tests. Unified Parkinson's Disease Rating Scale motor scores and error rates were assessed using paired *t*-tests. Given the exploratory nature of the study Bonferroni corrections were not applied to multiple comparisons. Statistical significance was set at 0.05. Effect size (ES) was reported as partial *η* ^2^ values.

## 3. Results

**Figure 2:**
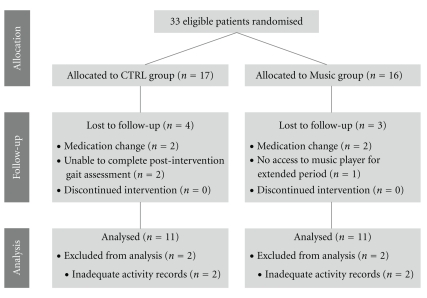
Study flow chart.

Thirty-three PD patients were enrolled into the study; complete data collected from twenty-two subjects were used in final analysis (see Figure 2 for study flow chart). Subject demographics and clinical characteristics at baseline are provided in Table 1. 

### 3.1. The Effect of Task on Gait Parameters

**Figure 3:**
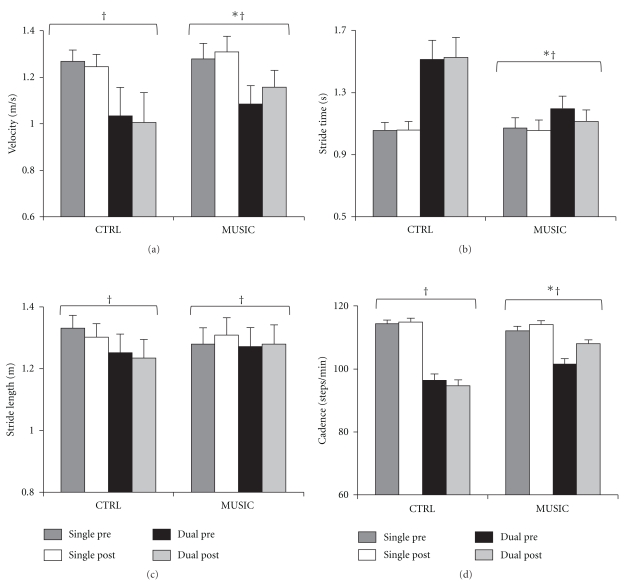
Effect of intervention on gait parameters in single and dual task conditions. Effect of intervention on (a) velocity, (b) stride time, (c) stride length, and (d) cadence during single task and dual task conditions, pre- and post-intervention amongst CTRL and MUSIC groups (means and standard errors). *Significant effect of Time. † Significant effect of Task.

Descriptive statistics for all outcome measures are provided in Tables 2 and 3 for the CTRL and MUSIC, subjects, respectively. A main effect for Task indicated that CTRL subjects walked more slowly (*F*[10] = 7.749, *P* = .019, ES =.437; Figure 3(a)), with shorter strides (*F*[10] = 8.661, *P* = .015, ES = .464; Figure 3(c)), and decreased cadence (*F*[10] = 5.021, *P* = .049, ES = .344; Figure 3(d)), and had a tendency towards increased stride time (*F*[10] = 3.279, *P* = .100, ES = .247; Figure 3(b)) in the dual task condition. Similarly the MUSIC group walked with decreased velocity (*F*[10] = 25.413, *P* = .001, ES = .718; Figure 3(a)), stride time (*F*[10] = 8.646, *P* = .015, ES = .464; Figure 3(b)), stride length (*F*[10] = 30.325, *P* < .001, ES = .752; Figure 3(c)), and cadence (*F*[10] = 10.688, *P* = .008, ES = .517; Figure 3(d)) when walking in the dual task condition.

### 3.2. The Effect of Intervention on Outcome Measures

The 13-week intervention period did not have an effect on the outcome measures of the CTRL group (*P* > .05). In addition, Time-by-Task interactions did not reach significance (*P* > .05), indicating that the changes to gait parameters observed among the CTRL group following the 13-week intervention period were consistent across single and dual task conditions. In contrast, the MUSIC group demonstrated a significant increase in velocity (*F*[10] = 17.474, *P* = .002, ES = .636; Figure 3(a)) and cadence (*F*[10] = 11.629, *P* = .007, ES = .538; Figure 3(d)), and a decrease in stride time (*F*[10] = 7.740, *P* = .019, ES = .436; Figure 3(b)) following the 13-week intervention. Time-by-Task interactions approaching significance were observed for velocity (*F*[10] = 3.756, *P* = .081, ES = .273; SINGLE = 2.34% increase; DUAL = 6.68% increase; Figure 3(a)), stride time (*F*[10] = 4.417, *P* = .062, ES = .306; SINGLE = 1.42% decrease; DUAL = 6.86% decrease; Figure 3(b)), and cadence (*F*[10] = 4.654, *P* = .056, ES = .318; SINGLE = 1.74% increase; DUAL = 6.86% increase; Figure 3(d)) suggesting that the intervention had a differential effect on the magnitude of improvements to single and dual task gait performance amongst the MUSIC group. A nonsignificant main effect of Time for error rate (*P* > .05) indicated that the MUSIC group did not alter their error rate for the secondary arithmetic task following the intervention.

Following the 13-week intervention, the MUSIC group demonstrated a statistically significant improvement in UPDRS (III) score (*t*[10] = 4.045, *P* = .002; 5.6 point reduction); the CTRL group also experienced a decrease in UPDRS score; however, the improvement for the CTRL group failed to reach significance (*t*[10] = 1.128, *P* = .286; 1.8 point reduction).

### 3.3. Feasibility of Intervention

Each group experienced 9 falls during the intervention period. Two separate subjects experienced falls in each of the groups. The falls did not occur during physical activity. Compliance with the intervention was good; two subjects in the MUSIC group took a one-week break due to scheduling conflicts. MUSIC subjects reported mean walk duration of 115 ± 28 minutes per week with their individualised music, with 90 minutes per week being the suggested weekly walk duration. The overall mean walk duration during the intervention did not differ significantly between groups *t*(20) = 1.073, *P* = .296; CTRL = 185 ± 139 minutes per week; MUSIC = 138 ± 47 minutes per week). 

Subjects rated the experience of exercising to their own programmed music as 9.0 on a 10-point rating scale. In addition, ten out of the 11 subjects in the MUSIC group indicated that they were comfortable enough with the design and the operation of the personal music player that they would consider using it again. One subject experienced difficulties with the complexity of the music player and the earphone cables but managed to complete the intervention. All MUSIC subjects indicated that they liked the music that was selected for them, with three subjects requesting partial or complete music changes during the intervention. Furthermore, nine of the 11 MUSIC subjects reported that they walked more than they expected to as a result of using the music, with two subjects reporting that the music had motivated them to try other exercises outside of walking. When asked to describe the benefits, if any, derived from walking with music responses, they said, “I walked with an increased pace, even after turning the music off”, “I stood taller and swung my arms more”, “I walked smoother, it was more even”, “I had an improved emotional state”, “exercising was less monotonous”, and “the music provided extra motivation to exercise”.The majority (9/11) of patients assigned to the MUSIC group did not report any unfavourable effects of the intervention; however, one subject reported some cramping in their thigh at night after walking while another patient reported feeling more tired during the intervention. Nine out of the 11 subjects in the MUSIC group planned to continue exercising with music in the future.

## 4. Discussion

This study investigated the feasibility and efficacy of using cadence-matched salient music as a gait training tool for PD patients. In agreement with our hypothesis, the findings of this study indicate that PD patients who trained for 13 weeks with a music program improved gait performance. In addition, the high compliance rate to the intervention and the limited reports of adverse effects provide the possibility that salient music may be a beneficial compliment to a walking activity intervention for use in the Parkinsonian population. 

The CTRL group maintained gait performance across all measures and task conditions following the intervention. Conversely, and in agreement with previous intervention studies using rhythmic auditory cues [14, 19, 21, 23] subjects who had walked with cadence-matched, salient music demonstrated improved gait performance following the intervention. In the single task condition the MUSIC group demonstrated marginal improvements in gait velocity, cadence, and stride time post-intervention. The same pattern of improvement was observed with larger relative magnitude in the dual task condition. This differential effect of the intervention by task was supported by strong two-way (Time × Task) interactions for the measures of velocity, stride time, and cadence. Consistent with prior reports that rhythmic auditory cues tend to be more effective in improving temporal as opposed to spatial parameters of gait [35–37], stride length remained largely unchanged in both conditions following the intervention. The enhanced gait performance demonstrated by the MUSIC group was accompanied by a significant improvement in motor symptom severity following the intervention. Further analysis revealed that the observed improvements did not reflect posture and gait items of the UPDRS, but instead they were confined to the components of the UPDRS that contribute to the akinesia subscale (Q23–26). 

The number of falls experienced during the intervention period was consistant across the two groups. Furthermore, the reported falls occurred whilst patients were carrying out activities of daily living within their homes and not whilst patients were engaged in the walking intervention. This implies that safety was not further compromised by the intervention for the group who listened to music whilst walking. In addition, the use of a personal music player to provide the individualised playlist during the intervention was well accepted by the subjects as indicated by patient responses in the Post-intervention Questionnaire. The subjective ratings of improved gait performance and/or emotional state were reported by all subjects in the MUSIC group, providing additional insight to the benefits provided by the intervention and providing further support for the feasibility of using salient music as a practical and sustainable cueing strategy. Adverse effects of the intervention reported by the patients in the MUSIC group are consistent with common symptoms of PD [38–40] and therefore may reflect the symptom profile of the disease rather than any detriment imposed by the walking program. 

The mechanism through which cadence-matched, salient music improves gait performance and motor symptoms in PD patients is equivocal. The improvement in uncued gait and inconsistent changes in stride time variability do not support the frequently postulated suggestion that rhythmic auditory cues act as an external pacemaker. One alternative explanation in agreement with the work of Sacrey et al. [41] is that the music may have enhanced gait performance through increasing the patients' affective arousal. The arousal potential of the music was intentionally high, with pieces selected based on familiarity and enjoyment. The improvement in dual task gait performance for the MUSIC group following a period of training with music may be representative of benefits of dual task training. Practicing two tasks concurrently allows the improvement of task-coordination skills [42]; therefore if listening to music is a cognitively demanding task, as we have suggested [24], it becomes possible that the intervention may inadvertently provide dual task training.

## 5. Limitations

This study included a comparatively small sample of PD subjects; furthermore the subjects were relatively heterogeneous between groups when considering baseline disease severity (UPDRS (III)). The limited sample size used in this study limits both the statistical power of the statistical analyses as well as the generalizability of our findings to the wider patient community. In addition, the heterogeneity of the sample groups at baseline necessitates cautious interpretation of the findings, with the possibility that the observed improvements in gait performance and symptom severity demonstrated by the MUSIC group could simply represent a placebo effect, or in the case of symptom severity a regression to the mean. Based on similar results following training with rhythmic auditory tones [22], we propose, as an alternative, that these preliminary results support the notion that a walking program accompanied by cadence-matched, salient music can improve single and dual task gait performance amongst PD patients with mild to moderate disease severity. 

A second major limitation of this study was that the CTRL group continued with regular activities as opposed to completing the walking program without music. This design decision reflected the characteristics of the local PD community who are actively encouraged to maintain a regular walking routine. As such, our available sample of convenience was comprised of individuals who included walking as part of their regular routine. Therefore, we are unable to elucidate whether the improvements to gait observed in this study reflect walking with music or the documented benefits of sustained walking. Modified Baecke questionnaire scores, however, indicated that each group had similar activity levels prior to the study, with the majority of subjects being regular walkers. In addition, the two groups spent a comparable amount of time walking during the intervention period (CTRL = 2289 ± 1696 minutes; MUSIC = 1791 ± 647 minutes; *t*(20) = .908, *P* = .375). These data lend support to the possibility that the improvements to gait performance observed in the MUSIC group were due to the accompaniment of cadence-matched, salient music. Future studies should incorporate a walking control group to verify this conjecture. Despite the limitations of the current study the findings should be used to direct the design of larger-scale randomized control trials investigating the efficacy of incorporating cadence-matched salient music into a gait training program. Further investigations should also utilise planned follow-up assessments to establish the optimal training frequency and duration necessary to retain improvements to gait performance and symptom severity. 

In conclusion, our results indicate that the use of cadence-matched, salient music to accompany walking is a feasible and enjoyable intervention for use amongst patients with mild to moderate PD. Further research is warranted to elucidate the effect of training with a salient music accompaniment on gait performance.

## Figures and Tables

**Table 1:** Subject demographics and clinical characteristics at baseline.

Characteristic	CTRL	MUSIC	*P*
No. of subjects	11 (4 Lethbridge)	11 (3 Lethbridge)	
Sex (M/F)	5/6	6/5	.67
Age (yrs)	67.0 (8.1)	64.1 (4.2)	.33
Disease duration (yrs)	4.5 (3.3)	6.4 (4.2)	.23
MMSE	28.4 (1.8)	29.3 (1.3)	.20
Hoehn and Yahr	2.1 (0.4)	2.3 (0.4)	.43
Baecke score	10.2 (4.8)	8.9 (3.8)	.48
UPDRS (III) score	20.4 (5.0)	25.5 (9.3)	.13

Values are mean (standard deviation) for continuous variable and number for nominal variables.

MMSE: Mini Mental Status Examination; UPDRS:Unified Parkinson's Disease Rating Scale. MMSE, Hoehn and Yahr, and UPDRS scores were measured with subjects on anti-Parkinsonian medication.

**Table 2:** Summary of descriptive statistics and change scores for the CTRL group for outcome measures pre- and post-intervention.

Measure							*T*	Ta	*T*× Ta
		Single			Dual				
	PRE	POST	change	PRE	POST	change			
Velocity (m/s)*	1.27(0.16)	1.25(0.17)	0.02	1.03(0.41)	1.01(0.42)	0.03	.232	.019	.869
Stride time (s)	1.06(0.11)	1.06(0.13)	0.002	1.51(0.88)	1.53(0.90)	−0.01	.876	.100	.906
Stride length (m)*	1.33(0.13)	1.30(0.14)	0.03	1.25(0.20)	1.23(0.20)	0.02	.167	.015	.394
Cadence (steps/min)*	114(11.7)	115(13.1)	−0.62	96.3(32.8)	94.7(35.5)	1.64	.786	.049	.459
Error rate (%)	NA	NA	NA	13.4(19.7)	16.2(19.4)	−2.80	.631	NA	NA
UPDRS (III) score	20.4(5.03)	18.6(7.38)	1.82	NA	NA	NA	.286	NA	NA

PRE and POST values are presented as mean (standard deviation). Change values are PRE minus POST and are presented as mean. *A negative change value indicates an improvement in measure.

*T* : Time; Ta: Task.

**Table 3:** Summary of descriptive statistics and change scores for the MUSIC group for outcome measures pre- and post-intervention.

Measure							*T*	Ta	*T* × Ta
		Single			Dual				
	PRE	POST	change	PRE	POST	change			
Velocity (m/s)*	1.28(0.22)	1.31(0.22)	−0.03	1.09(0.26)	1.16(0.24)	−0.07	.002	.001	.081
Stride time (s)	1.07(0.07)	1.06(0.07)	0.02	1.20(0.15)	1.11(0.08)	0.08	.019	.015	.062
Stride length (m)*	1.36(0.17)	1.37(0.18)	−0.01	1.27(0.20)	1.28(0.21)	−0.01	.524	.000	.887
Cadence (steps/min)*	112(7.86)	114(7.85)	−1.95	102(12.0)	108(7.76)	−6.53	.007	.008	.056
Error rate (%)	NA	NA	NA	13.2(12.8)	7.42(9.50)	5.76	.155	NA	NA
UPDRS (III) score	25.5(9.28)	19.9(9.05)	5.55	NA	NA	NA	.002	NA	NA

PRE and POST values are presented as mean (standard deviation). Change values are PRE minus POST and are presented as mean. *A negative change value indicates an improvement in measure.

*T* : Time; Ta : Task.
